# Tryptophan degradation in irritable bowel syndrome: evidence of indoleamine 2,3-dioxygenase activation in a male cohort

**DOI:** 10.1186/1471-230X-9-6

**Published:** 2009-01-20

**Authors:** Gerard Clarke, Peter Fitzgerald, John F Cryan, Eugene M Cassidy, Eamonn M Quigley, Timothy G Dinan

**Affiliations:** 1 Department of Psychiatry, University College Cork, Cork, Ireland; 2 Alimentary Pharmabiotic Centre, University College Cork, Cork, Ireland; 3 Department of Pharmacology & Therapeutics, University College Cork, Cork, Ireland; 4 Department of Medicine, University College Cork, Cork, Ireland

## Abstract

**Background:**

Irritable bowel syndrome (IBS) is a common disorder that affects 10–15% of the population. Although characterised by a lack of reliable biological markers, the disease state is increasingly viewed as a disorder of the brain-gut axis. In particular, accumulating evidence points to the involvement of both the central and peripheral serotonergic systems in disease symptomatology. Furthermore, altered tryptophan metabolism and indoleamine 2,3-dioxygenase (IDO) activity are hallmarks of many stress-related disorders. The kynurenine pathway of tryptophan degradation may serve to link these findings to the low level immune activation recently described in IBS. In this study, we investigated tryptophan degradation in a male IBS cohort (n = 10) and control subjects (n = 26).

**Methods:**

Plasma samples were obtained from patients and healthy controls. Tryptophan and its metabolites were measured by high performance liquid chromatography (HPLC) and neopterin, a sensitive marker of immune activation, was measured using a commercially available ELISA assay.

**Results:**

Both kynurenine levels and the kynurenine:tryptophan ratio were significantly increased in the IBS cohort compared with healthy controls. Neopterin was also increased in the IBS subjects and the concentration of the neuroprotective metabolite kynurenic acid was decreased, as was the kynurenic acid:kynurenine ratio.

**Conclusion:**

These findings suggest that the activity of IDO, the immunoresponsive enzyme which is responsible for the degradation of tryptophan along this pathway, is enhanced in IBS patients relative to controls. This study provides novel evidence for an immune-mediated degradation of tryptophan in a male IBS population and identifies the kynurenine pathway as a potential source of biomarkers in this debilitating condition.

## Background

Irritable bowel syndrome (IBS) is one of the most commonly diagnosed functional bowel disorders [1] and, while reports of its prevalence vary [2], a reasonable estimate from the literature indicates that it occurs in 10–15% of the population [3]. A clear understanding of disease pathophysiology still eludes researchers [4], although it is increasingly being categorised as a disorder of the brain-gut axis [5]. Visceral hypersensitivity has received the most attention as a putative biological marker but its use as a diagnostic marker is controversial [6] and other less invasive candidates need to be validated. Accumulating evidence points to the involvement of both central and peripheral serotonergic systems in disease symptomathology [7,8]. Serotonin is involved in regulating gastrointestinal tract (GIT) secretion, motility and perception [9,10] and it's role in the regulation of mood is also well documented [11]. Although much of the attention in the literature has tended to focus on the conversion of tryptophan to serotonin, the kynurenine pathway actually represents the dominant metabolic cascade in mammals, accounting for over 95% of the available peripheral amino acid [12]. Of late the realisation that the metabolites so generated possess inherent biological activity has contributed to increased interest in a previously understudied area.

The first rate limiting step in the pathway involving the conversion of tryptophan to kynurenine is catalysed by either the ubiquitous indoleamine 2,3-dioxygenase (IDO) or tryptophan 2,3-dioxygenase (TDO) which is localised to the liver [13]. Kynurenic acid, produced from kynurenine in a sidearm of the cascade, is a NMDA receptor antagonist at physiogical concentrations through its competitive blockade of the glycine co-agonist site [14]. Quinolinic acid, produced along an alternative branch of the pathway, has excitotoxic properties due to potent activation of NR2A and NR2B NMDA receptor subtypes and its ability to generate free radicals independently of receptor activation [14]. The activity of IDO is intertwined with the state of the immune system and it can be potently induced by cytokines such as interferon-γ, thus providing a mechanism through which either central or peripheral immune activation can impact on serotonergic and glutamatergic functionality [15]. The activity of TDO can be increased by l-tryptophan and it's analogues via an allosteric binding site and is competitively inhibited by some common indoleamines including tryptamine [13]. In addition, the ability of corticosteroids to induce TDO is also well established [16]. As low level immune activation has been implicated in IBS [5], the kynurenine pathway of tryptophan metabolism may represent an key mediator of the physiological consequences of altered immunoregulation in the disease.

This study was performed to assess tryptophan and its kynurenine pathway metabolites in IBS. Although IBS is more prevalent in females than males, there is a sizeable and underappreciated prevalence in male sufferers [2]. Moreover because tryptophan levels are known to fluctuate significantly over the course of the menstrual cycle [17] we confined our study to a male patient population. Neopterin, a sensitive marker of immune status [18], has previously been identified as a marker of disease activity in inflammatory bowel disease (IBD) [19]. We also measured this immune marker in this study to identify the source of any proposed kynurenine pathway alterations.

## Methods

### Subjects

Twenty-six healthy male control subjects (Mean age 32.2 years, range 20–51 years) and ten male IBS patients (Mean age 47.5, range 28–65 years) participated in the study. Patients were diagnosed with IBS based on Rome II criteria, and controls were free from physical illness. The study was powered to detect differences in analyte levels at the 0.05 level and was approved by an ethics committee (Research Ethics Committee of the Cork Teaching Hospitals). All subjects provided written, informed consent and were recruited from a university database of IBS patients. The database comprised of people who had either attended gastroenterology clinics at Cork University Hospital or had responded to direct advertisement on the university campus or local newspaper regarding participation in IBS research. Patients also underwent a physical examination and a full review of clinical history. Healthy controls were recruited from the complement of staff affiliated to the University College Cork and its teaching hospitals. All patients and healthy subjects were within 10% of ideal body weight and were free of serotonergic medications. Two of the patients (20%) and four of the controls (15.4%) were classified as smokers. All individuals were free of transient illness within 2 weeks of study participation. Venous blood samples were collected in the morning in tubes with EDTA as anticoagulant after an overnight fast. The samples were centrifuged (3500RPM, 15 min, 4°C) and the plasma stored at -80°C until processing.

### Reagents

HPLC grade acetonitrile, acetic acid and perchloric acid were obtained from Alkem/Reagecon (Cork, Ireland). All other reagents, including the reference metabolites and internal standard, were obtained from Sigma (Dublin, Ireland) unless otherwise stated.

### Plasma preparation

Plasma samples were spiked with internal standard (3-Nitro l-tyrosine) prior to being deproteinised by the addition of 20 μl of 4 M perchloric acid to 200 μl of sample. Samples were centrifuged at 14000 RPM on a Hettich Mikro 22R centrifuge (AGB, Dublin, Ireland) for 15 minutes at 4°C and 100 μl of supernatant transferred to a HPLC vial for analysis.

### Preparation of standards

Stock solutions of each standard were prepared in HPLC grade water. Working dilutions were prepared from the stock standards, aliquoted in suitable vials and stored at -80°C until required for analysis. Standards were acidified with 20 μl 4 M perchloric acid prior in injection onto the HPLC system.

### HPLC equipment

The HPLC system consisted of a Waters 510 pump (Waters, Dublin, Ireland) 717plus cooled Autosampler (Waters), a Hewlett Packard 1046A fluorescent Detector (Agilent, Dublin, Ireland), a waters 996 photodiode array (PDA) detector (Waters), a waters bus SAT/IN module (Waters) and a Croco-Cil column oven (Waters). System components were used in conjunction with Waters Empower software. All samples were injected onto a reversed phase Luna 3 μm C18 (2) 150 × 2 mm column (Phenomenex, London, England), which was protected by Krudkatcher disposable pre-column filters (Phenomenex) and SecurityGuard cartridges (Phenomenex).

### HPLC conditions

The HPLC conditions were modified from a previously described method [20]. Briefly, the mobile phase consisted of 50 mM acetic acid, 100 mM zinc acetate with 3% (v/v) acetonitrile and was filtered through Millipore 0.45 μm HV Durapore membrane filters (AGB) and vacuum degassed prior to use. Compounds were eluted isocratically over a 30-minute runtime at a flow rate of 0.3 mls/min after a 20 μl injection. The column was maintained at a temperature of 30°C and samples/standards were kept at 8°C in the cooled autoinjector prior to injection. The fluorescent detector was set at an excitation wavelength of 254 nm and an emission wavelength of 404 nm. The PDA detector was set to scan between 210–400 nm with chromatogram extraction at 330 nm.

### Analyte identification and quantitation

L-tryptophan and its metabolites (kynurenine, kynurenic acid) were identified by their characteristic retention times as determined by injections of standards which were run at regular intervals during sample analysis. Concentrations were determined using analyte:internal standard peak height ratios. Results were expressed as μmol/L of plasma.

### Neopterin ELISA

Neopterin levels in the plasma samples were measured using a commercially available quantitative enzyme-linked immunosorbent assay (ELISA) system (IBL, Hamburg, Germany). Samples were assayed in duplicate according to the manufacturers' instructions. The absorbance was read at 450 nm on a Biotek Synergi plate-reader (Mason, Cork, Ireland) and the results calculated from a 4-parameter logistics curve generated using Gen5 software (Mason). The assay is sensitive to 0.7 nmol/L with an intra-assay coefficient of variation of 3.6% and an intra-assay coefficient of 7.6% at the 7.4 nmol level.

### Data Analysis

All data are reported as mean ± SEM. Student's t-tests were employed to determine differences in analyte levels. Bonferroni corrections for multiple t-tests were employed as necessary. Correlation analysis between neopterin measures and the kyn:trp ratio was performed using a Pearson product-moment correlation. Due to the possibility of their being an influence of age on the results, we used age as a covariate assessed by analysis of co-variance (ANCOVA).

## Results

### Tryptophan and metabolites in IBS patients

**Figure 1:**
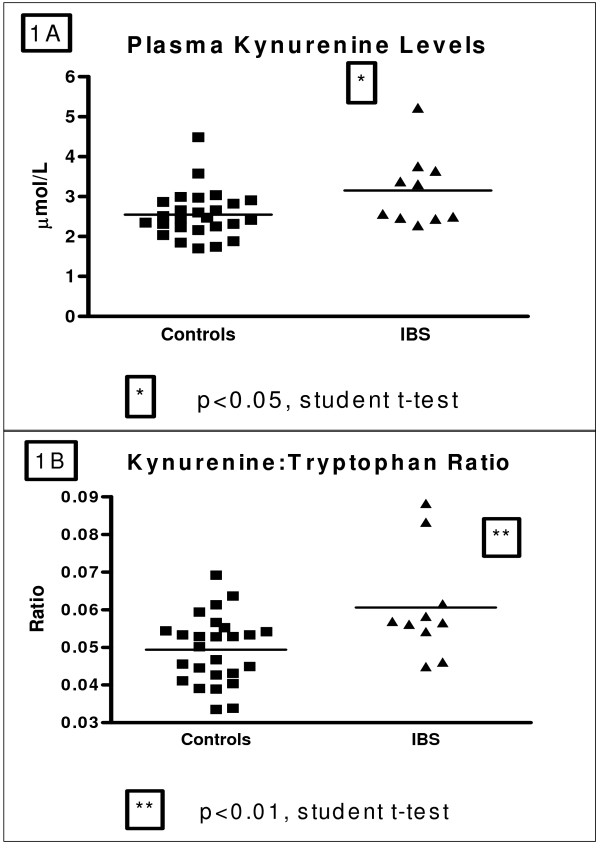
**A/B: (A) Plasma kynurenine Levels (μmol/L) in healthy controls and male IBS patients and (B) Kynurenine:tryptophan ratio in healthy controls and male IBS patients**.

**Figure 2:**
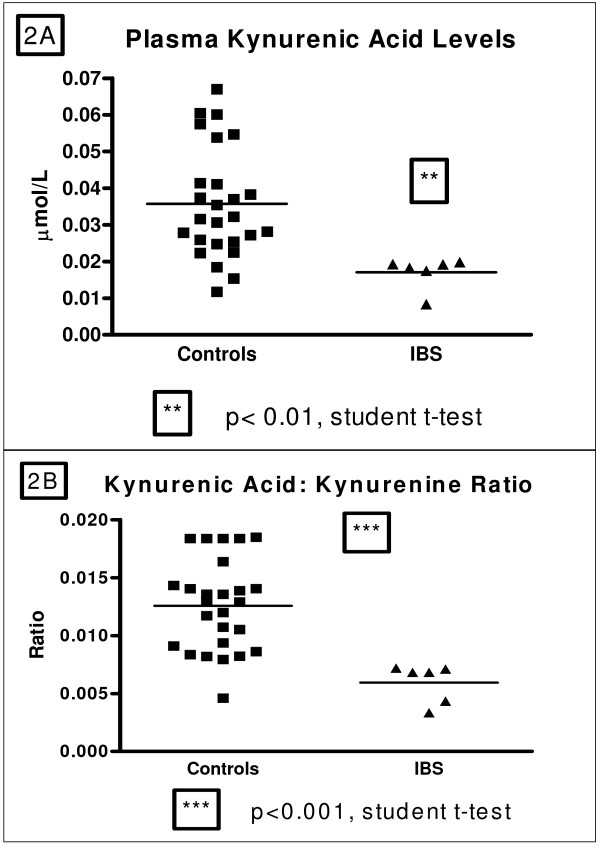
**A/B: (A) Plasma kynurenic acid levels (μmol/L) in healthy controls and male IBS patients and (B) Kynurenic acid:kynurenine ratio in healthy controls and male IBS patients**.

There were no differences in plasma l-tryptophan levels between IBS patients and control subjects (53.37 ± 2.28 vs. 53.32 ± 3.01 μmol/L, p = 0.9826). As shown in figure 1A, l-kynurenine levels were increased in IBS patients relative to control subjects (3.16 ± 0.29 vs. 2.55 ± 0.12 μmol/L; t = 2.348, df = 34, p < 0.05) as was the l-kynurenine:l-tryptophan ratio (0.06058 ± 0.0045 vs. 0.04938 ± 0.0017; t = 2.820, df = 34, p < 0.01) (Figure 1B). Moreover, as shown in Figure 2A kynurenic acid concentrations were decreased in IBS patients compared with control subjects (0.017 ± 0.002 vs. 0.036 ± 0.003 μmol/L, t = 2.979, df = 30, p < 0.01) as was the kynurenic acid: l-kyurenine ratio (0.005938 ± 0.0006 vs. 0.01258 ± 0.0007; t = 4.138 df = 39, p < 0.001) (Figure 2B). It should be noted that, for technical reasons, kynurenic acid could only be quantified in 6 of the 10 IBS patient samples.

### Neopterin Levels

**Figure 3:**
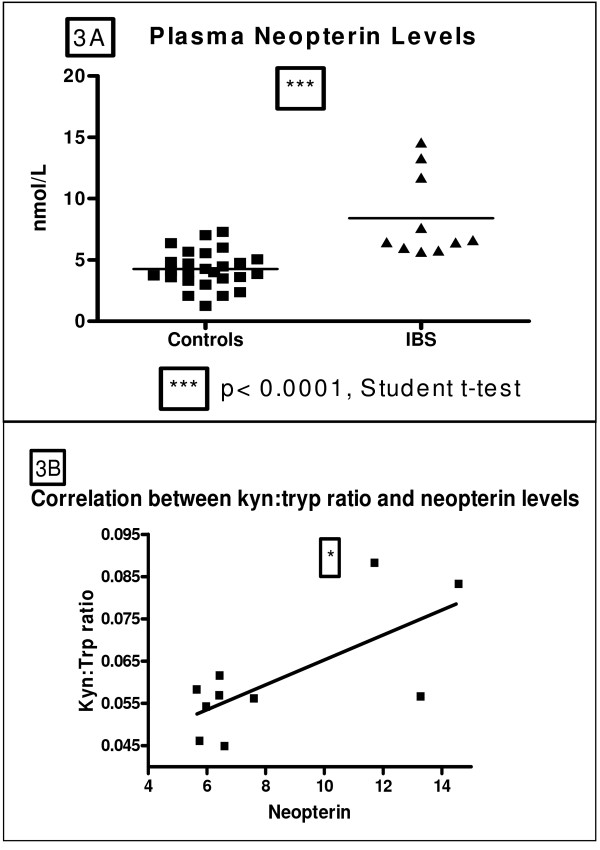
A/B: (A) Plasma neopterin levels (nmol/L) in healthy controls and male IBS patients.(B)Correlation between neopterin levels and the kyn:tryp ratio (Pearson product-moment correlation, *p < 0.05, r = 0.7055).

Neopterin Levels were increased in IBS patients relative to controls (8.406 ± 1.080 vs. 4.250 ± 0.2941 nmol/L, t = 5.129, df = 34, p < 0.0001) (Figure 3A).

### Correlation results

There was a significant correlation between neopterin measures and the L-Kynurenine:l-tryptophan ratio (Figure 3B, P = 0.023, r = 0.7055).

### ANCOVA Results

ANCOVA analysis revealed no significant effect of age on any of the parameters tested.

## Discussion

Although some studies have examined kynurenine pathway metabolites in relation to gastrointestinal function [21], there is a paucity of reports on tryptophan metabolism in an IBS population. One of the most interesting findings in this study was that both plasma kynurenine levels and the kynurenine:tryptophan ratio were significantly increased in the male IBS cohort compared to healthy control subjects. Although there is considerable group overlap between tryptophan and kynurenine, the ratio is considered a more appropriate indicator of the degree of tryptophan degradation along this pathway than absolute measurement of its components [22]. The fact that both kynurenine and the ratio were increased in the subjects tested is compelling evidence of increased activity of the enzymes responsible for tryptophan breakdown in this patient population and has many possible ramifications. The decreased peripheral availability of tryptophan could impact on central uptake and affect serotonin synthesis [13]. Indeed this hypothesis is supported by acute tryptophan depletion studies, whereby, through a dietary intervention which limits the central availability of tryptophan, a state of reduced serotonin synthesis, metabolism and release is induced. Moreover, these studies have been recently employed to demonstrate a dysfunctional serotonergic system in IBS [3,23]. It is, however, difficult to relate acutely altered tryptophan levels in those studies to the apparent chronic disturbances in the tryptophan metabolic pathway noted here. While we failed to detect any alteration in actual tryptophan levels in the current studies, which may lead one to suggest that the relevance of the increased breakdown may be physiologically questionable, the impact of increased kynurenine levels needs to be factored in to the interpretation of these results. Kynurenine and tryptophan enter the central nervous system (CNS) via the same transport mechanism [14] and the increased competition at this transporter should restrict tryptophan uptake into the CNS. The consequences of increased peripheral tryptophan breakdown also need to be considered, since it is unlikely that an increased rate of breakdown would solely impact on central serotonin production *per se*.

In the gastrointestinal tract serotonin has a role in visceral sensation and pain transmission [24] and pharmacological agents acting at these receptors, including alosetron (5-HT_3 _antagonist) and tegaserod (5-HT_4 _agonist), have been shown to modulate intestinal transit [25]. Whether the alterations described in this study could have a physiologically relevant impact on enteric serotonergic signalling has not yet been confirmed but remains an interesting possibility. Whilst it is conceivable that the increased kynurnenine:tryptophan ratio originates in the increased activity of hepatic TDO, the alternative scenario of increased IDO activity is equally, if not more, plausible. The elevated neopterin levels in the IBS cohort strongly suggest that IDO is the main enzymatic player. Although the majority of the neopterin measurements were below the 10 nmol level, which is considered to be reliably indicative of a disease state [26], they do confirm, at the very least, a low level immune activation in the male IBS group. Of interest is that the statistical difference between the groups is much greater for this measure than for the tryptophan indices. This may be due to the fact that while neopterin is considered to be a general marker of immune activation, IDO is only activated by specific immune agents [13]. While we cannot conclusively say that there is an increased central tryptophan degradation from our peripheral measures our conclusions are in line with that of Saito *et al*. [16] who previously demonstrated increased IDO activity both peripherally and centrally following systemic immune stimulation. Because elevated IDO activity could further impact on both central and peripheral serotonergic transmission through its ability to metabolise serotonin [27], if IDO is activated, the knock-on consequences for serotonergic signalling are greater than if it is TDO-mediated. In this regard, the sensitive and reliable measurement of interferon-γ levels, which were below the limit of quantitation (LOQ) of the analysis method employed in the current samples (unpublished data), would be most useful in future studies to further assess immune activation and confirm IDO activation in IBS cohorts [26]. Our results also show decreased plasma kynurenic acid levels as well as a decrease in the kynurenic acid:kynurenine ratio. This ratio is sometimes termed the neuroprotective ratio and essentially indicates a decreased conversion of kynurenine to kynurenic acid [28]. In the current studies, kynurenic acid could only be determined in six out of ten of the IBS population. However, a coincidental increase due to low statistical power is unlikely due to the decrease in both kynurenic acid and the kynurenic acid:kynurenine ratio.

It is tempting to suggest that these data represents a shunting of tryptophan away from the kynurenic acid branch and towards the preferential formation of the neurotoxic metabolite but we did not measure quinolinic acid in this study and, therefore, cannot conclusively say that this is indeed the case. The ability of quinolinic acid to activate both central and peripheral glutamatergic transmission could greatly impact on gastrointestinal function.

While these results suggest that the routine measurement of tryptophan pathway metabolites in IBS patients may provide biomarkers of the disease state a number of study limitations need to be considered before coming to this conclusion. The exclusively male patient population in this study was relatively small in size so that further subgroup analysis of the results according to disease status, type or symptom severity was not practical. Consequently, we cannot assign the observed alteration to diarrhoea predominant, constipation predominant or alternating symptoms. Neither can we say that the alterations are indicative of an active or quiescent phase of the disease. The study could also be criticised for its reliance on male IBS samples. However, it should be pointed out that, although IBS is more prevalent in the female population, a significant number of male patients present with the condition [2]. In addition, the measurement of tryptophan levels in a female population can be complicated by variations over the course of the menstrual cycle [17]. In any case, the results strongly suggest that a future expanded study should be conducted that would allow a more detailed analysis of the biomarker potential of the tryptophan metabolites.

## Conclusion

In summary, we have identified alterations in key indices of tryptophan metabolism in a male IBS population which are attributable to immune activation. We suggest that when used in conjunction with neopterin measures, they may be useful as a potential biomarker panel in IBS.

## Abbreviations

(IBS): Irritable Bowel Syndrome; (IDO): Indoleamine 2,3-Dioxygenase; (HPLC): High Performance Liquid Chromatography; (UV): Ultra-Violet; (ELISA): Enzyme-linked Immunosorbent Assay; (TDO): Tryptophan 2,3-Dioxygenase; (NMDA): N-methyl-D-aspartate; (BBB): Blood Brain Barrier; (CNS): Central Nervous System; (KAT): Kynurenine Amino Transferase; (SERT): Serotonin reuptake transporter.

## Competing interests

The authors declare that they have no competing interests.

## Authors' contributions

GC performed the HPLC and ELISA assays and analysed the data. PF recruited the patients and collected the samples. TGD, EMQ, PF and EC participated in the conception and design of the study. GC, TGD, EMQ, PF, & JFC interpreted the results and wrote the manuscript. All authors have read and approved the final manuscript.

## Pre-publication history

The pre-publication history for this paper can be accessed here:

http://www.biomedcentral.com/1471-230X/9/6/prepub
